# Assessment of the Suitability of Ceramic Waste in Geopolymer Composites: An Appraisal

**DOI:** 10.3390/ma14123279

**Published:** 2021-06-14

**Authors:** Ismail Luhar, Salmabanu Luhar, Mohd Mustafa Al Bakri Abdullah, Marcin Nabiałek, Andrei Victor Sandu, Janusz Szmidla, Anna Jurczyńska, Rafiza Abdul Razak, Ikmal Hakem A Aziz, Noorina Hidayu Jamil, Laila Mardiah Deraman

**Affiliations:** 1 Department of Civil Engineering, Shri Jagdishprasad Jhabarmal Tibrewala University, Rajasthan 333001, India; jprraj2017@gmail.com; 2 Frederick Research Center, P.O. Box 24729, Nicosia 1303, Cyprus; 3 Center of Excellence Geopolymer and Green Technology (CEGeoGTech), Universiti Malaysia Perlis (UniMAP), Arau 02600, Perlis, Malaysia; rafizarazak@unimap.edu.my (R.A.R.); ikmalhakem@unimap.edu.my (I.H.A.A.); noorinahidayu@unimap.edu.my (N.H.J.); lailamardiahderaman@gmail.com (L.M.D.); 4 Department of Civil Engineering, Frederick University, Nicosia 1036, Cyprus; 5 Faculty of Chemical Engineering and Technology, Universiti Malaysia Perlis (UniMAP), Arau 02600, Perlis, Malaysia; 6 Department of Physics, Czestochowa University of Technology, Dabrowskiego 69, 42-201 Częstochowa, Poland; nmarcell@wp.pl; 7 Faculty of Materials Science and Engineering, Gheorghe Asachi Technical University of Iasi, 71 D. Man-geronBlv., 700050 Iasi, Romania; sav@tuiasi.ro (A.V.S.); 8 Faculty of Mechanical Engineering and Computer Science, Częstochowa University of Technology, 42-201 Częstochowa, Poland; j.szmidla@imipkm.pcz.pl (J.S.); annajurczynska@vp.pl (A.J.)

**Keywords:** ordinary Portland cement (OPC), construction and demolition wastes (CDW), porcelain tile polishing residue (PPR), geopolymer, ceramic powder waste (CPW), waste clay brick powder (WBP), ceramic sanitary-wares wastes (CSW)

## Abstract

Currently, novel inorganic alumino-silicate materials, known as geopolymer composites, have emerged swiftly as an ecobenevolent alternative to contemporary ordinary Portland cement (OPC) building materials since they display superior physical and chemical attributes with a diverse range of possible potential applications. The said innovative geopolymer technology necessitates less energy and low carbon footprints as compared to OPC-based materials because of the incorporation of wastes and/or industrial byproducts as binders replacing OPC. The key constituents of ceramic are silica and alumina and, hence, have the potential to be employed as an aggregate to manufacture ceramic geopolymer concrete. The present manuscript presents a review of the performance of geopolymer composites incorporated with ceramic waste, concerning workability, strength, durability, and elevated resistance evaluation.

## 1. Introduction

Over several years, OPC has been extensively employed globally as a binder to manufacture concrete and an assortment of edifice materials. However, it is a scientifically proven fact that the contemporary production process is highly energy intensive with necessitating elevated temperatures of about 1450 to 1550 °C and, more importantly, not an ecobenevolent one since it causes grave environmental pollution through the emission of a substantial quantity of greenhouse gases (GHG) [1,2,3,4,5]. Unfortunately, OPC production alone is blamed for virtually 6 to 7% of total CO_2_ emissions—a primary GHG, as estimated by International Energy Agency (IEA) [6]. In fact, among total GHG, around 65% of the dilemma of global warming is assigned to the emission of CO_2_ alone. Incredibly, it is predicted that the global mean temperature could increase by around 1.4 to 5.8 °C over the next one hundred years [7]. One ton of production of OPC emits an almost equal quantity of CO_2_ in the atmosphere [8,9]. Worldwide, in the existing backdrop of emissions of CO_2_ mediating the climate alteration, the mean sea level is also supposed to rise because of, on the one hand, the thawing of permanently frozen ground on the earth and, on the other hand, the thermal expansion of water, in addition to frequently occurring natural calamities may cause titanic losses to the economy [10,11]. Furthermore, emitted GHG, viz., CO_2_, SO_3_, and NOX, from the OPC industries can cause damage to the fertility of the soil and acid rain [12]. Generally speaking, the industrial consumption of natural raw materials is estimated as 1.5 tonnes for one tonne of Portland cement production [13,14]. Key natural raw materials, such as limestone, coal, clay, etc., are running out swiftly. In order to resolve these problems, scientists, researchers, engineers, and the industrial workforce are ceaselessly dedicating many endeavours in order to find new progressive and innovative building materials and substitute binders. Globally, ceramic industries are generating enormous amounts of wastes every year, creating huge landfills that are harmful to environmental dilemmas. Statistically, the world’s ceramic tile production is greater than 10 million square metres per year [15]. An estimation has been made that roughly 15 to 30% of this production is regarded as unutilized wastes [15] forming great heaps. It has been proven [16,17,18,19] that ceramic is strongly resistant to biological degradation processes. Ceramic is an appropriate supplementary cementing material for enhancing the mechanical properties and durability properties of concrete since ceramic is found enriched with silico-aluminium crystalline materials [20,21]. However, the reuse of ceramic waste in the construction industry is still in its primary stage in a negligible quantity [22]. It is worth mentioning that millions of tons of natural, agricultural, and industrial wastes, viz., waste ceramic powders, cement dust, fly ash, byproducts generated from coal and oil burning, bottom ash, palm oil fuel ash, bagasse ash, discarded tires, marble, and crushed stone, are disposed of in Malaysia every year [19,23,24,25,26,27,28] and responsible for severe environmental concerns, namely the leaching out of perilous substances and air pollution. China, as the world’s greatest consumer and producer of ceramics, generates over one million tonnes of all kinds of ceramic wastes, such as tiles, blocks, pan forms, etc., which are filling useful land spaces or piled up each year. This is why it is desirable to investigate the possibilities to use waste of ceramic as a replacement for natural aggregates such as gravel or sand. Senthamarai and Manoharan [16] have studied the attributes of concrete with electrical ceramic waste aggregate, whereby they observed that the compressive, splitting tensile, and flexural strengths of ceramic waste coarse aggregate concrete were inferior to traditional concrete. Analogously, a generation of construction and demolition wastes (CDW) of ceramic and the bricks are found increasingly all over the world due to the reconstruction and renovation activities of older structures, which make up approximately 45% of the total CDW [29,30,31]. Senthamarai and Manoharan [16] and Hussein et al. [19] have reported that around 30% of the total ceramic waste is produced by the ceramic industry. The researchers are taking an ever-increasing interest in exploring new alumina-silicate materials. Various attention-grabbing endeavours have been made for recycling and reusing these CDW-containing ceramic wastes and bricks as pozzolanic materials along with them [32,33,34,35]. Astonishingly, it has been accounted that more than one million tons of ceramic wastes are generated each year in China alone [36]. Although the abovementioned residue can be reused in a few ceramic material productions, most of it is discarded in open landfills [37]. However, ceramic residue wastes must possess certain prerequisites to be activated, such as higher solubility in the basic alkali medium, and its precursors must be rich in alumina and silica. Advantageously, the ceramic wastes can be effortlessly separated from dumps because they are not attached to gypsum, cement, or other binders, which makes them easy to reuse and facilitates their valorisation. The peculiar feature of biodegradation of ceramic residues, such as ceramic sanitary-ware wastes, is found to be long-lasting, even up to 4000 years. For these reasons, its potential application and valorisation as a precursor to manufacture novel optional binders with low CO_2_ footprints through Geopolymerization remain an attractive alternative. Research on the applications of porcelain tile polishing residue (PPR) in OPC materials is scarcely conducted [38,39,40,41]. However, the investigations have demonstrated the impacts of the PPR supplement on OPC mortars and that the substitution of OPC with PPR partly contributes to the hydration process. The said effect was assigned to the pozzolanic activity, filler impact, and heterogeneous nucleation caused by the residue. The blending of PPR in OPC production is a well-recognized strategy for recycling the said waste. Another application of this residue, taking into consideration its chemistry, is in geopolymeric binder manufacturing due to sufficient concentrations of alumina and silica, which lowers the cost of geopolymer carbon footprint impact. Interestingly, wastes of ceramic sanitary-wares products, such as washbasins, lavatories, bidets, etc., are not only the result of the renovation of buildings but also due to product rejection due to manufacturing defects during production, such as breakage, shape deformation, and/or trivial defects, such as finishing, etc., which often crop up while firing the pieces and affect the physical, chemical, and aesthetic attributes of the ultimate product. Following data revealed by Baraldi et al. [42], the global production of sanitary wares is estimated to have escalated by 61.3% in a decade, increasing from 216.6 to 349.3 million pieces produced in 2004 and 2014, correspondingly. Additionally, it has been noted by Medina et al. [43] that 5 to 7% of the pieces are discarded because they have defects. This simply means that roughly 20 million of pieces can be regarded as waste. Moreover, more ceramic damaged in the course of storage, transportation, construction, and the renovation of houses increases the abovementioned percentage of waste generation. Furthermore, the disposal procedures are turning out to be more costly on account of the ever-rising limit to restrictions on landfills. All the above problems have compelled industries to look for solutions to these predicaments and turn to recycling such wastes to transform them into useful products. Looking at some recent studies on ceramics, how ceramic wastes can be employed in manufacturing building materials for construction and infrastructure industries has been investigated. For example, ceramic wastes can be reused in concrete production, although the majority of research has been focused on the analysis of the mechanical and durable attributes of ceramic as a binder or fine and/or coarse aggregate for the partial replacement of OPC [44]. The course of action of employing wastes of ceramic has been studied, but the echelons of waste which were utilized efficiently for this objective were very small and no dedicated investigation has accounted for the methodical characterization of the synthesis and high-temperature characteristics of geopolymer pastes employing ceramic wastes as raw material. The chemistry of ceramic with extremely crystalline aluminosilicate made it feasible to be employed to develop good-quality geopolymer mortars and concrete. Sun et al. [36] have applied one more kind of ceramic residue to attain geopolymeric cement and estimated its mechanical behaviour while taking into account the effect of elevating the temperature. After 28 days, the composition attained a compressive strength of 71 MPa, which augmented to 75 Mpa following exposure at 1000 °C for 2 hrs. Nevertheless, the ceramic residue was obtained from municipal solid wastes, a tile mix, pan forms, and blocks which were treated before. At first, the ceramic residue was washed ultrasonically to remove impurities, viz., scraps of paper, plastic, organic matter, and metals. Afterwards, the dried ceramic wastes were crushed and pulverized through a ball mill for 45 min. The PPR possesses a pozzolanic nature and its chemistry is enough to facilitate the production of geopolymeric binders. Hence, the acceptable outcomes were obtained by Sun et al. [36]. Initial investigations have demonstrated homogeneity in the granulometric and chemical composition of this kind of residue, which was gathered once every seven days for up to 90 days [41]. The incorporation of ceramic in manufacturing ceramic geopolymer concrete not only provides a systematic solution to the problem of its disposal but also assists in addressing the environmental issues arising due to its piling up. Likewise, the cost savings associated with its disposal in landfills can be made, and the substitution of natural aggregates from restricted natural resources will help the conservation of natural wealth in addition to safeguarding the environment. This means that the reuse of said wastes as aggregate in concrete might prove to result in a win-win situation. In other words, on the one hand, the solution to the consumption of ceramic industry waste can be achieved through its well-organized disposal, while, on the other hand, this contributes to a more sustainable construction material. In addition to this, ceramic geopolymer concrete also provides relief to the great dilemma of global warming with its user and ecofriendly nature, as well as being indicative of an affordable, durable, and sustainable construction material for the future.

In the year 1979, the term “geopolymer” was coined by J. Davidovits to describe complex inorganic polymers made up of alumina-silicates and possessing a 3D structure network united by several tetrahedral units of SiO_4_ and AlO_4_ linked with oxygen produced through an exothermic process of “geopolymerization” at low temperature and atmospheric pressure [9]. This means that geopolymers are produced from aluminosilicate minerals of either geological origin or industrial byproducts as precursors, and they are synthesized in a highly alkaline medium with alkali activators. The process of geopolymerization is analogous to natural rock-forming “geosynthesis”. 

RILEM, the International Union of Laboratories and Experts in Construction Materials, Systems and Structures, has considered geopolymers as a class of alkali-activated materials exercising acronyms such as C–A–S–H and N–A–S–H to designate the hydration of the geopolymeric system, which develops a gel structure when having higher and lower calcium systems. Geopolymerization is the exothermic reaction of solid aluminosilicates with hydroxide and silicate solutions of alkali that encourages the dissolution of Al^+3^ and Si^+4^ ions from starting materials and, subsequently, the formulation of an alkali-activated gel demonstrating good-quality mechanical and durable characteristics in the hardened phase [45,46]. Geopolymers based on fly ash, blast furnace slag, or natural pozzolan are found to be quite cost effective. Geopolymers demonstrate not only six times lower energy costs but also, significantly, nine times lower emissions of CO_2_ (carbon dioxide) gas in comparison to the present OPC system. The abovementioned alkali-activation of industrial byproducts significantly lowers the call for long-established natural raw materials and aggregates in cement and concrete, which immediately mitigates CO_2_ emissions, landfilling, and energy consumption, too [47]. Furthermore, their user-friendly nature and exhibition of outstanding attributes in relation to strength, durability, thermal and chemical resistance, etc., are significant. They seem to be a potential replacement material for OPC due to some other excellent properties, such as high initial strength and lower acoustic and thermal conductivities, which are identical or even superior to OPC [48,49].

## 2. Ceramic Powder Waste [CPW]

Ceramic powder is the principal waste found to be generated by the ceramic industry in the form of discarded dust during dressing and polishing ceramic items. Quite often, CPW is partly consumed on site for refilling the excavation pit. Ceramic wastes are nonbiodegradable and require spacious lands to dispose of them. For those reasons, finding an innovative way to recycle this waste and then to blend it for the development of new construction materials for infrastructure through geopolymer technology can be helpful to conserve not only limited natural resources but also the environment. However, hitherto, the development of diverse geopolymers as ecowelcoming construction materials by incorporating said waste materials has hardly ever been explored.

Preceding research has [20] unearthed that wastes from the ceramic industry possess pozzolanic properties, and hence they can be employed to make concrete with enhanced strength and durability. Following Pacheco et al. [22], it is possible to manufacture mortars exhibiting superior strength and durability performance through the replacement of conventional sand with CPW. Geopolymers with the replacement of fine natural aggregates by CPW were found to be efficient with a little lower absorption of water. This kind of water permeability of such geopolymers implies that the changeover of conventional sand by CPW can be considered as a brilliant option.

The pozzolanic behaviour of clay brick powder WBP and CPW was explored previously to substitute OPC in manufacturing of cement paste and concrete [50,51]. Furthermore, Rakhimova et al. [52] have examined the potential of employing WBP in producing geopolymer cement containing 20% augmentation of GGBFS. The activation of WBP and CPW is dependent on the Na concentration, i.e., the percentage of Na_2_O, silica modulus (SiO_2_/Na_2_O), and curing techniques. In line with their report [52], compressive strengths ranging from 0 to 120 MPa were recorded with a silica modulus value as 1.5, a 5% Na concentration, and a high temperature of about 90 to 95 °C, along with curing for 3 h, and a conclusion was made that red clay brick waste may substitute for GGBFS up to 60%. Geopolymer mortar incorporating WBP was manufactured by Reig et al. [31] using dissimilar concentrations of alkali activator. The use of 7% Na concentration, a silica modulus value of 2, and a high temperature of 65 °C along with curing for one week has resulted in a compressive strength gain of up to 50 MPa.

Despite gaining optimistic benefits, the high-temperature curing has escalated the cost of the paste/mortar, noticeably hindering its practical application and restricting its utilization in cast-in situ constructions [53].

## 3. Ceramic Waste as Aggregate

Additionally, industrial wastes can be employed as recycled aggregate. Since the quality, quantity, size, and kind of recycled aggregates to be utilized impact strongly on the durability and mechanical attributes of the concrete being designed, they need to be characterized correctly in accordance with Silva et al. [54] and Behera et al. [47]. The monitoring of Elgaali and Elchalakani [55] has revealed that recycled concrete strength was influenced strongly by the quality of aggregates. The abovementioned conclusion was found to be corroborated in diverse studies, whereby ceramic waste has been utilized as a recycled aggregate in PC systems. Medina et al. [56] have noted that an enhancement in context to mechanical characteristics in concrete can be made by the application of recycled aggregate derived from CSW—waste of ceramic sanitary ware. The ceramic tiles are a special sort of CDW found to be massively generated in the east of Spain. The research work of Alves et al. [57], in which fine ceramic aggregates were used, witnessed shrunken mechanical properties as compared to the reference concrete. The report of Stock [58] has revealed that the Spanish industry has manufactured 420 out of the total production of 11,913 million square metres of tiles in the year 2013, attaining the fifth position for top manufacturing nations after China, Brazil, India, and Iran. The said Spanish manufacturing was about 3.5% of total global production. The investigation of the potential application of gigantic quantities of ceramic tile waste as both a precursor and recycled aggregate in preparing geopolymer mortars is a fascinating and attractive option for tile and cement industries, together with interest in recycling as well as the production of more sustainable edifice materials.

The primary investigations on the geopolymerization of waste of ceramic sanitary-wares (CSW) were fruitfully carried out by Reig et al. [59], who examined the optimum mix proportions of sodium hydroxide/sodium silicate and the impact of supplements of Ca(OH)_2_ on the fresh behaviour, mechanical strength, and microstructure of the designed binders. The conclusion was made that mortars activated by solutions blended with 4.5 wt.% of Ca(OH)_2_ have displayed the best workability with good-quality compressive strength benefits of 21 and 27.5 MPa after a curing of 3 and 7 days at 65 °C, correspondingly. Additionally, it was monitored that the addition of Ca(OH)_2_, has proven to be essential for geopolymerization kinetics, which prompts a vast interest in advance analyses on the impact of a variety of Ca sources on the mechanical attributes and microstructure of geopolymer mortars incorporated with CSW. In this context, the preceding investigations have thrown light on this crisis and analysed the effect of supplements, viz., calcium aluminate cement (CAC), Portland cement (PC), or Ca(OH)_2_, on the reactivity and characteristics of blended geopolymer systems by using diverse precursors [60,61,62]. In general, they concluded that these admixtures boost the reaction kinetics of the system, and the contribution was made to the development of mechanical strength of the specimens. Unambiguously, the impact of Ca(OH)_2_ on geopolymerized ceramic materials was explored by Reig et al. [63]. Although wastes of porcelain stoneware tiles were productively activated and mortars with compressive strengths in the vicinity of 30 MPa with 7 days curing at 65 °C were obtained, no activation happened to occur in the absence of Ca-hydroxide, and swift setting took place when 5% of the residue was substituted with the said source of Ca. Furthermore, the research investigated the effect of the concentration of alkali activator and dosage of Ca on the activation course of porcelain stoneware tiles. The conclusion unearthed that mortars manufactured with an unvarying molar ratio of calcium and sodium (MCa) represented an identical setting time. When both the SiO_2_ concentration and MCa were constant, the mortars demonstrated a linear increase concerning the compressive strength with the Na concentration. The review of Shi et al. [62] shows that supplements of CAC have been monitored to augment the mechanical characteristics and reactivity of geopolymer binders. The findings were corroborated in a study by Reig et al. [64], whereby CAC provided evidence for the acceleration of the course of activation of red clay brick waste, escalating the compressive strength by 68.6% and 165.7% in the mortars containing 20 wt.% and 40 wt.% of CAC with a curing for 7 days at 65 °C, in that order. Both investigations have revealed the same opinion about the case of systems blending up to 20 wt.% cement; the pattern for CAC hydration diverges from that usually monitored in water, and the aluminium and calcium from CAC are taken up in the geopolymeric alumina-silicate gel formulated, so that the typical CAC hydrates that emerged through standard water hydration were not identified in the geopolymer specimens.

## 4. Ceramic Industry Sludge 

Sludge obtained from the ceramic industry is also considered to be waste material which encompasses kaolinite—a metakaolin hydroxylated form. It is a kind of solid waste produced by a few ceramic companies at the end of the wastewater management process. The ceramic industrial waste region currently overruled the eco-logical influence of this form of waste by a decrease from the restricted natural resources of the kaolinite. It may be employed as a raw material through substitution as metakaolin in the construction sector [65,66].

Many studies have made it known that the efficacy of the reaction of pozzolanic nature or the kinetics of geopolymerization is linked to the dehydroxylation extent to raw materials [67,68,69]. Consequently, the application of entirely dehydroxylated amorphous-type metakaolin boosts the performances of concrete greater than the partly dehydroxylated type. The path of dehydroxylation begins with the proton movement from a hydroxyl group to a different hydroxyl group, followed by the H_2_O formulation molecule and ends with the removal of the water molecule. The competency of this advance depends on the calcination period [70,71]. In accordance with Ptáček [72], a great loss in mass was monitored in the TGA pattern at around a temperature of 500 °C, which is due to the exit of hydroxyl- groups enclosed in the mineral as water molecules. In reality, 4OH groups depart the deposit to form two particles of H_2_O, leaving two particles of oxygen as per the equation portrayed below [73]:

4OH^−1^ → 2H_2_O + 2O^−2^ (1)

In the structure of the crystal, the changes during the entire calcination vary markedly, as many intrinsic parameters count the early material’s crystallinity. The Stoch Index (IK), which is based on the XRD pattern, is based on material surface imperfections [73]. The greater the IK value, the more mineral surface flaws. Furthermore, if the value of (IK) is less than 0.7, the mineral has a well-thought-out network, while if (IK) is higher than a unit, it is considered that the mineral has a distorted network. 

The ceramic industrial sludge used in the study was more crystalline as compared to kaolin. Conceptually, a geopolymeric cement may be produced by any type of alkali solution. However, Na_2_CO_3_ and NaOH or KOH—are most prevalent in the usage of its manufacturing. The alkaline cations impact geopolymerization kinetics in several ways. The presence of an ion of sodium boosts the dissolution of the source of alumina-silicate because of its smaller size by the formulation of the geopolymer through additional regimented forms. Nevertheless, the existence of ions of potassium is inclined to enhance the extent of polycondensation due to their bulky size, which supports the development of larger silicate, inducing an inferior aptitude to the formulation of a crystalline type. The use of a water glass combination and potassium hydroxide thereby increases the mechanical characteristics of geopolymers [74].

## 5. Characteristics of Ceramic Waste Incorporating Geopolymer Composites 

### 5.1. Flow/Slump

Huseien et al. [75] have unveiled the findings of the flow test of the synthesized geopolymers with flow values of 16.5, 18.5, and 23 cm when the quantity of ceramic was augmented from 50 to 60 to 70%, in that order, in comparison with the control specimen which represented a 13.2 cm flow diameter. One more cause to boost the workability of mortars is to enhance the waste ceramic powder (WCP) quantity and trim down the GBFS content by substituting it with FA and WCP. This influences the rate of chemical reaction kinetics [53,76,77] and augments the plasticity of a mix which enhances the workability of geopolymers. Furthermore, they monitored that an enhancement in fly ash (FA) content has declined the workability of geopolymers because of the higher water adsorption of FA with a porous structure [78,79,80]. 

Hwang et al. [81] have manufactured strongly workable WBP incorporating geopolymer paste and WCP-blended geopolymer paste by using waste red clay brick powder (WBP) and waste ceramic powder (WCP), in that order. The slump flow benefits were found to range from 235 to 285 mm and 245 to 300 mm for the mixtures of WBP incorporating geopolymer paste and WCP-blended geopolymer paste, correspondingly. The WBP incorporating geopolymer paste displayed an inferior workability to the WCP-incorporated geopolymer paste because of its larger specific surface area and higher porosity.

### 5.2. Setting Time

Huseien et al. [82] have also examined mortars with the maximum WCP content, which has taken the longest time of 92 min to set. A sharp drop off in the setting time was reported when the amount of WCP was lessened and the quantity of GBFS was boosted. Enhancement in the quantity of FA has affected the setting time substantially. Both the early and final setting times were found to be escalated with the boosted level of FA substituting GBFS. That means that the higher setting time was reported. Additionally, it has been in line with the fact that the more the GBFS quantity is in the mortar, the quicker the setting rate is [79,83].

### 5.3. Compressive Strength

**Figure 1:**
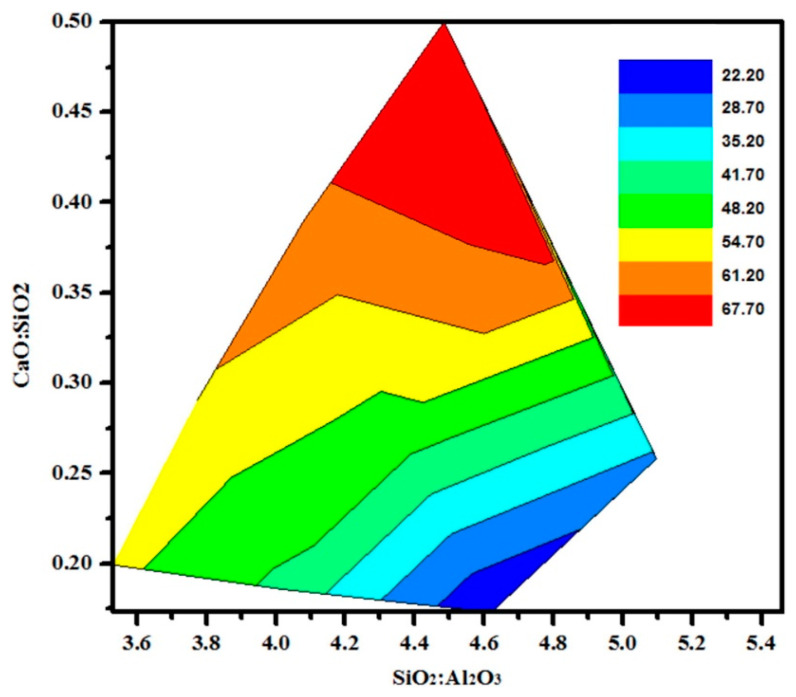
Effect of varying Ca:Si on the compressive strength of WCP-based geopolymer.

Huseien et al. [75] have worked on the compressive strength of geopolymers and found it to vary inversely with the escalating quantity of WCP from 50 to 70%, whereby the strength was found to be trimmed down from 70.1 to 34.8 MPa after 28 days, correspondingly. The negative impact of the inferior quantity of Ca and higher amount of silica was assigned to this fall [84,85,86]. This led to the development of lesser C-A-S-H gels and that is why it mitigated the strength of geopolymers. This is because WCP contains more than 70% silica with a larger particle size than GBFS, which affected the strength development of the samples of geopolymers manufactured with a higher quantity of WCP. Furthermore, the samples manufactured with a higher amount of WCP have also obtained 81%, 94%, and 97% strength at the ages of 28, 56, and 90 days, in that order (See Figure 1 [75]). Additionally, comparable findings were recorded by Rashad [13] who monitored a dwindle in the compressive strengths with a rising amount of FA in the matrix of geopolymer. The second aspect is the lower rates of reaction kinetics of WCP and FA compared with GBFS, which was dissolved partly.

Reig et al. [87] have investigated the application of ceramic tile waste (TCW) as both a precursor (TCWP) and a recycled aggregate (TCWA) to attain a binding mix through the course of geopolymerization. The findings for the compressive strength of the TCWP containing geopolymer mortars cured at 65 °C for 3 days chiefly rely upon the kind of material utilized as the recycled aggregate and vary based on the total (w’/b) ratio of the mixture or, to some extent, with particle size. The RCB incorporating geopolymer mortars, whose recycled aggregate demonstrated the highest water absorption, have displayed the best benefits, whereas the least strength development is encountered with the aggregate having the minimum water absorption values (CSW).

Hwang et al. [81] have reported the values for compressive strength from 36 to 70 MPa for the geopolymeric paste with curing at ambient temperature. The best possible value for strength was recorded from the WBP incorporating geopolymer paste and WCP-blended geopolymer paste with 30% GGBFS, while the lowest strength value was obtained from the WCP-blended geopolymer paste with 10% GGBFS. High-strength WBP incorporating geopolymer paste and WCP-blended geopolymer paste mixtures were produced utilizing higher percentages of WBP and WCP, in that order, under ambient temperature condition. 

Huseien et al. [88] have studied WCP-based geopolymers incorporating FA and found an influence on their development concerning compressive strength. The gain in context to strength fell because the quantity of FA escalated from 0 to 40%. However, the geopolymers manufactured with 40% FA as a substitution for GBFS obtained adequately higher compressive strength (of about 45.9 MPa), which could allow this yield to be applied and utilized in quite a lot of applications in the construction industry. Villaquirán-Caicedo and Mejía de Gutiérrez [89] have also reported that the compressive strength found improved upon utilizing an exposure temperature from 25 to 1200 °C because of the densification and crystallization of the geopolymer gel. At 900 °C, all pastes have demonstrated a drop in compressive strength. The referred loss of strength at 900 °C is found to be linked to the augmented surface energy of the gel coming into existence due to the loss of water from the surface and the appearance of smaller pores in the structure of the gel of geopolymer, causing its structure collapse. In consequence of the restructuring of the gel and the formulation of new crystalline phases at 1200 °C, the compressive strength in every system is augmented. These benefits are analogous to those recorded and employing conventional silicates [90].

Reig et al. [91] have estimated the impact of dissimilar quantities and sources of Ca [CAC, PC, and Ca(OH)_2_] on the mechanical characteristics of the alkali activation of the waste of ceramic sanitary ware (CSW) and ultimately concluded that their mechanical attributes are enhanced substantially with Ca supplement. In contrast, 40.06 and 64.41 MPa were achieved in mortars manufactured with 6 wt.% Ca(OH)_2_ and 10 wt.% PC, in that order, and 56.65 and 70.69 MPa were attained in those containing 10 and 15 wt.% CAC, correspondingly (each of them was cured at 65 °C for 7 days). The said findings were similar to those observed before in Reig et al. [91] for porcelain stoneware tile waste incorporating geopolymers, whereby a value up to 36 MPa was met within mortar specimens blended with 5% Ca(OH)_2_, cured for 7 days at 65 °C.

The investigations by Garcia Lodeiro et al. [60], whereby FA and PC blended pastes (30 wt.% PC) were activated with NaOH/Na_2_SiO_3_ solutions, resulted in 35 MPa strength after 365 days at room temperature. Likewise, a value of 33 MPa was noted in a hybrid alkaline cement containing 60 wt.% clinker and 40 wt.% incinerator waste (a mixture of fly ash plus bottom ash), activated with a mixture of CaSO_4_, Na_2_SO_4_, and water, along with curing for 28 days. The benefits demonstrated by the CSW/CAC-blended geopolymer mortars vary from those formerly noted by Fernandez-Jimenez et al. [92] for metakaolin/CAC-blended geopolymer pastes manufactured with 20% CAC, whereby strength values of around 13 MPa were found after curing for 20 h at 85 °C. The strength of the CSW/CAC geopolymer mortars were enhanced substantially with up to 20 wt.% CAC replacements and typically declined with further additions. The abovementioned behaviour could be assigned to the hindrance in the CAC hardening progression monitored previously by Pastor et al. [93] when hydrating CAC with strongly alkaline solutions of NaOH. Here, with the CSW-CAC-activated system under the studied conditions, they found that the interaction between NaOH in the activating solution and CAC particles escalated with high a CAC content, which, therefore, slowed down the hydration of CAC. Additionally, this behaviour diverges from that previously monitored for red clay brick waste containing geopolymer and CAC-incorporated systems, whereby values for compressive strength accelerated gradually with the supplement of CAC (values around 40, 60, 70, and 92 MPa in the geopolymer mortars that enclosed 10, 20, 30, and 40 wt.% CAC, correspondingly, with curing at 65 °C for a week).

Ariffin et al. [94] have studied the compressive strength of mortar with a dissimilar percentage of ceramic aggregate as river sand substitution. After a curing period for one day, every sample attained the strength of 40MPa. The controlled specimen exhibited an augmentation of 10 to 32% from 3 days to one week of age, whereas the cubes containing 50% and 100% quantity of ceramic as sand substitution represented an enhancement of 44 to 63%, correspondingly. A total of 100% of the ceramic-containing sample attained 80MPa compressive strength on the 7th day. This may be due to the higher content of SiO_2_, CaO, and Al_2_O_3_ in multiple amalgamated ash and ceramic aggregate, which provides the strong reaction of N-A-S-H and C-A-S-H of multiple incorporated ashes together with the alkaline solution in developing a higher strength to the geopolymer mortar.

Ramos et al. [95] have studied the influence of porcelain tile polishing residue on geopolymer cement, and for all compositions, an augmentation of compressive strength was confirmed with a curing time from 7 to 28 days. The synthesis of geopolymers incorporating waste, employing porcelain tile polishing residues as a raw material, encourages their application as cost-effective and lower energy materials for the construction industry. The application of PPR with metakaolin in manufacturing geopolymers can enhance the compressive strength by an average of 10%, resulting in a compressive strength greater than 70 MPa. The referred boost in strength is most likely due to the residue chemistry, which is formed by alumina and silica and represents an amorphous structure because of the attributes of ceramic tile processing.

Additionally, Zhang et al. [96] have monitored an enhancement in strength as to different curing times for geopolymers based on metakaolin and sodium hydroxide. They assigned this boost in mechanical resistance with curing time to the permanence of the process of geopolymerization. The concentration of NaOH showed no impact on the compressive strength, whereas the stronger concentration of the water glass was exhibited to be disadvantageous to compressive strength. 

Following Zhang et al. [96], surplus alkali could dissolve the only recently developed products of reaction again by breaking the chemical bonds, hence deteriorating the structure of the geopolymer. The silica content present in geopolymer cement is in line with the best mechanical resistance benefits for geopolymeric systems (between 45 and 55%), and alumina content should range between 22 and 28% to achieve the optimum results for mechanical strength.

Kuenzel et al. [82] have altered mortars with metakaolin-based geopolymeric materials into polycrystalline nepheline/quartz ceramics with a higher compressive strength of about 275 MPa and a higher Vickers hardness of around 350 MPa. The findings therein demonstrated that the geopolymers incorporating PPR also represented a higher hardness and that the PPR has contributed to enhancing this characteristic.

The greater hardness for compositions with 15% PPR are found to be about 300 MPa, which is attributed to their contents of silica and alumina (SiO_2_/Al_2_O_3_ ¼3.44) that outline the poly-sialate-disiloxo structure, considered by Davidovits [9] to be a more resistant microstructure. 

Although the ratios of SiO_2_/Al_2_O_3_ > 4 are not the most widespread, it is feasible to obtain superior mechanical benefits for a few compositions of geopolymer cement. Nevertheless, Sun et al. [36] have already manufactured geopolymer cement utilizing only ceramic residue treated earlier that was cured at 60 °C to examine its behaviour during firing at temperatures ranging from 60 to 1000 °C. The residue chemistry of 65% SiO_2_, 21% Al_2_O_3_ with its compressive strength of 70 MPa at 28 days, was very near to that found in the referred work and reinforces the binding effect of PPR for utilization in geopolymer cement.

When only the residue is employed, the percentage composition diverges from the references utilized in the factorial planning, intending to obtain a healthier performance. Taking into consideration the findings represented by Sun et al. [13], the curing temperature was 60 °C. The average strength was 16 MPa at 7 days of age, indicating the reactivity of the PPR residue by itself.

Yun-Ming et al. [78] have established that geopolymer powder has been fruitfully employed to manufacture one-part mixing geopolymers and geopolymeric ceramics. Finally, they concluded that the one-part mixing geopolymers only demonstrated an optimum compressive strength of 10 MPa after 28 days. The incessant formulation of the geopolymer matrix was evident after direct mixing with water. It was assumed that the presence of zeolite crystallites due to the blending with water diminished the compressive strength of the one-part mixing geopolymers.

Keppert et al. [79] have performed geopolymer synthesis employing two-poles-apart red-clay ceramic powders and a varying quantity of Na_2_O/SiO_2_ activator. The experimental findings demonstrated that despite the higher content of crystalline minerals and inferior concentration of amorphous matter, both examined precursors facilitate the production of geopolymers with agreeable mechanical characteristics. The measured values of compressive strength in relation to SiO_2_/Al_2_O_3_ and Al_2_O_3_/(Na_2_O + K_2_O) ratios in the manufactured geopolymer mixtures demonstrate that the consideration of a merely amorphous portion of the ceramics is suitable. The abovementioned benefit is in good harmony with the theoretical formula of geopolymeric materials and with the findings achieved for metakaolin-based geopolymers by other researchers, too.

The mechanical attributes, such as compressive strength of geopolymers, are often noted concerning SiO_2_/Al_2_O_3_, Al_2_O_3_/Na_2_O, and other ratios in the initial mix of activator and precursor [80,81,82,83,84,85,86,87,88,89,90,91,92,93,94,95,96,97,98,99,100].

Statistically, Lahoti et al. [83] have analysed the factors affecting the strength of MK based geopolymers and found the ratios of Si/Al and Al/Na to be the most effective parameters; the optimum values were recorded to be 1.7 to 2.2, i.e., 3.4 to 4.4, when articulated as SiO_2_/Al_2_O_3_, and 0.8 to 1.2, correspondingly. It is worth noting that the strength values met within the case of the MK-based geopolymer system of best possible composition were about twofold in comparison with the benefits attained for ceramic precursors in their study.

### 5.4. Split Tensile Strength

Huseien et al. [75] recorded that the tensile splitting strength was found to be influenced by the escalating quantities of WCP and represented an inferior strength of 2.68 MPa with 70% WCP content in comparison with 5.32 MPa attained for 50% amount of WCP and 5.84 MPa of a controlled specimen. An escalating amount of WCP led to the diminution of calcium quantity and lowered the rate of chemical reactions kinetics to yield the C-S-H gel [77,101,102]. An inverse relationship was encountered among the tensile splitting strength of geopolymeric materials and the fly ash quantity. When the amount of FA accelerated, the strength plunged. The account of Phoongernkham [103] unveiled that the strength was in enhanced in fly-ash-based geopolymers with the boost of calcium quantity where supplementary C–S–H and C–A–S–H gels co-existed with N–A–S–H gel. This elucidated the decline concerning strength with an increasing quantity of FA and WCP, as well as a cutback in the amount of GBFS. Ariffin et al. [94] have concentrated on the splitting tensile strength and found that at the age of 3 days, in samples with 50% and 100% ceramic aggregate as sand substitution, the tensile strength was augmented by around 18% and 31%, in that order, in comparison with the controlled specimens. The enhancement in the context of strength at 7-day curing was faintly boosted by roughly 8% and 17% higher than the controlled specimens.

### 5.5. Flexural Strength

The experiments of Huseien et al. [75] have shown that the flexural strength was found to decrease as the WCP quantity increased at the age of 28 days and were reported to be 10.12 MPa, 9.26 MPa, and 4.62 MPa for 50%, 60%, and 70% of the amount of WCP, correspondingly. The strength was found to drop when the quantity of FA was boosted in each echelon of WCP. The findings have signified that the flexural strength amplified on accelerating the amount of slag, which conforms with the results of preceding investigations [104]. The study by Ariffin et al. [94] demonstrated that the flexural strength of geopolymeric materials incorporated dissimilar percentages of ceramic aggregate replacing river sand. The findings led to a report that the curing period is fairly responsible for the momentous effect on the flexural strength. The strength augmentation after 7 days of 100% ceramic aggregate substitution is found to be about twofold. The strength growth of the controlled specimen for 3 days and 7 days is 3.28 MPa and 7.22MPa, in that order, in comparison with 4.27 MPa and 9.06 MPa of the specimens with 100% ceramic aggregate following 3 days and 7 days of curing.

### 5.6. Modulus of Elasticity

Research by Huseien et al. [75] has extended the values of MOE concerning geopolymer materials with dissimilar WCP quantity percentages such as 50%, 60%, and 70% at a curing time of 28 days. The results indicated that as the quantity of WCP-substituted GBFS escalated, the values of the MOE dropped. The enhancement in the percentage of WCP-substituted GBFS from 50%, 60%, and 70% led to a fall in the MOE figures from 16.3 GPa to 15.8 GPa to 7.4 GPa in comparison with 19.9 GPa reported with geopolymer materials (100% GBFS). The findings exhibit that a growing content of FA boosted the silicate quantity and mitigated Ca content. This, in turn, led to the plunge of the MOE of the geopolymeric materials manufactured. 

### 5.7. Water Absorption

A research study by Huseien et al. [75] on the topic of water absorption uncovered a direct proportionality with the quantity of WCP. The boost in the level of WCP-substituted GBFS augmented the water absorption from 8.5 to 8.6% and 11.2% with the swell in the amount of WCP from 50 to 60% and 70%, correspondingly, in comparison with the 5.6% noted for the controlled specimen. An escalation in the quantity of WCP and FA could influence the nonreacted and partly reacted particles to augment and lead to a structure which is more porous. In another investigation, Huseien et al. [88] illustrated the impact of FA substituting GBFS on water absorption of higher volume WCP-based geopolymer mortars at 28 days of curing. The records of water absorption disclosed a direct proportionality with the amount of FA. The boost in the FA level substituting GBFS amplified the water absorption from 7.3 to 8.5%, 9.4%, 9.7%, and 10.1% with the growing quantity of FA from 0 to 10%, 20%, 30%, and 40%, in that order. The water absorption of samples of geopolymer materials was affected by the pore structure of mortar specimens manufactured with a higher amount of FA. A boost in the quantity of FA could influence the nonreacted and partly reacted particles which led to an added porous structure. Nevertheless, each of the samples of geopolymeric materials exhibited a value of water absorption either equal to or less than 10% which could reach the acceptable standard for scores of use in the construction and infrastructure industries.

### 5.8. Carbonation Resistance

Depending on the ratio of FA to GBFS substitution, Huseien et al. have [88] shown the depth of carbonation of geopolymeric materials. The enhanced level of substitution of GBFS by FA from 0 to 10%, 20%, 30%, and 40% led to a boost in the depth of carbonation from 6.8 mm to 7.1 mm, 7.3 mm, 7.6 mm, and 8.2 mm, in that order. The abovementioned atypical conduct could be linked to a higher level of FA geopolymer materials matrix, which led to an added pore structure as the development of gel was confined by inferior Ca quantity and demonstrated an elevated permeability and porosity to water than in the control [105,106,107].

### 5.9. Acid Resistance

In the context of resistance to acid of geopolymeric materials, Huseien et al. [88] have explained the findings of residual strength at ages of 6 months and one year after immersion in a 10% H_2_SO_4_ solution. It was established that the residual strength is directly related to FA quantity. Following the immersion period, each of the geopolymeric materials exhibited a diminution in residual strength in comparison with a controlled specimen. Enhancement in the FA substituting GBFS from 0 to 10%, 20%, 30%, and 40% led to a swell in residual strength from 78.3 to 87.1%, 93.8%, 95.4%, and 99%, correspondingly, after exposure for 6 months. A comparable drift in benefits was also achieved within FA substituting GBFS in a higher WCP level following a one-year period. The Ca(OH)_2_ compound in the mortar reacted with the SO_4_^−2^ ion upon exposure of the geopolymer materials to sulphuric acid, forming gypsum (CaSO_4_ 2H_2_O). This phenomenon is accountable for the spreading out of the geopolymer matrix as well as supplementary cracking in the interior of samples. Much research [108,109,110] has found that augmented Si, Al, and Na quantities trimmed down the formulation of gypsum, therefore raising the durability of geopolymer materials.

### 5.10. Sulphate Attack

Huseien et al. [88] have replaced GBFS with FA in higher volumes of WCP-based geopolymer materials produced far above the ground performance mortars in severe environmental conditions. Following residual strength, XRD patterns as well as loss in mass, the resistance of geopolymer materials to an attack of sulphate improved as the amount of FA incorporating the said geopolymer materials was augmented. Geopolymer materials manufactured with 40% of fly ash demonstrated the maximum resistance amongst every mix. The inferior quantity of CaO in FA has contributed chiefly to putting a ceiling on the formulation of gypsum all through the immersion period.

### 5.11. Freeze–Thaw Cycles

Huseien et al. [88] have exemplified the impact of FA substituting GBFS on the residual compressive strength and percentage of loss in weight of geopolymer material samples in the open for freezing–thawing cycles. The findings have signified an inverse relationship among residual strength and loss in weight with a rising amount of FA. With an increased substitution of GBFS with FA, the drying shrinkage is found to be improved and exhibits inferior performance under freeze–thaw cycles, i.e., under aggressive environments. The number of voids may allow the ice development and smash the interlock among the particles [111].

### 5.12. Wet–Dry Cycles

The impact of FA substituting GBFS on the residual compressive strength of geopolymer materials was estimated through their exposure to wet–dry cycles. As shown by Huseien et al. [88], the strength loss of geopolymer materials was established as directly proportional to the quantity of FA present and the number of wet–dry cycles. This is because when the entire porosity escalates, there will be more added pores in the matrix, which provide supplementary chances for water to penetrate the matrix all through the wetting and drying cycles, which in turn leads to an increase in the inside and outside deterioration as well as durability loss with time [112]. Fortunately, every geopolymer materials represented much higher performance under wet–dry cycle checks.

## 6. Thermal and Elevated Temperature Study 

### 6.1. Thermal Conductivity

**Figure 2:**
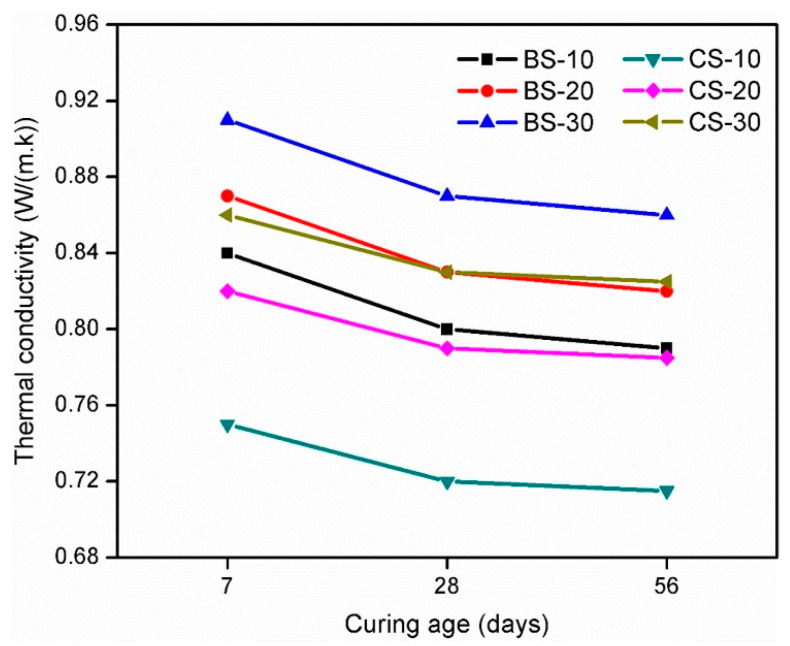
Thermal conductivity.

Thermal conductivity (TC) is a physical attribute of hardened paste which influences heat transfer by conduction throughout the toughened paste [113]. Thermal conductivity is influenced by the microstructure of hardening consolidated paste and the thermal properties of its constituents. A hard-bitten paste with high thermal conductivity shows evidence far above the ground values for heat transfer and energy consumption [114]. In the study of Hwang et al. [81], The values of thermal conductivity of the WBP incorporating geopolymer paste and WCP-blended geopolymer paste specimens were gauged at 7, 28, and 56 days of curing. The benefits of the examination of thermal conductivity are portrayed in Figure 2.

The greatest thermal conductivity was encountered for the hardened paste with 7-day curing and exhibited a declining tendency with an escalating curing period. Along with the augmented curing age, the quantity of extremely conductive free water was decreased by either an alkali activation reaction or evaporation from alkali-activated paste (AAP). Furthermore, with rising age, the formulated geopolymer gels such as C–S–H, C–A–S–H, and N–A–S–H are ever-increasingly dehydrated. Hydrated gels turn out to be dehydrated on account of the evaporation of free water, and air voids take the place of water. The thermal conductivity of AAPs having noteworthy free water and hydrated gels will be higher than that of AAPs with minimum free water and dehydrated gels because the thermal conductivity of water is 25-fold greater than the thermal conductivity of air [115,116,117,118]. Generally, the WBP incorporating geopolymer paste mixtures displayed higher results for thermal conductivity during the investigation than the WCP-blended geopolymer paste mixtures. There was a superior course of gelation in the WBP incorporating geopolymer paste mixtures than in the WCP-blended geopolymer paste mixtures, which subsequently provided the previously high compressive strength and a denser microstructure. Uysal et al. [119] formerly accounted that the thermal conductivity of concrete improved with an enhancement in compressive strength and a denser microstructure. On the other hand, the WBP incorporating geopolymer paste mixtures have shown notably higher benefits for thermal conductivity at early curing times, probably due to the higher porosity of WBP leading to its absorbing a huge quantity of water, which is extremely thermal conductive [120]. Following one-week-long curing, the benefits for thermal conductivity plunged at a steeper rate, as the water was consumed either during geopolymerization or was evaporated. The thermal conductivity of both types of referred mixtures escalated with boosting the quantity of GGBFS. This may be attributed to reasons such as: (1) as the amount of GGBFS augments, hydrated gels will be formulated, and as a consequence, a denser microstructure will be developed, and (2) as GGBFS is denser than FA, the supplement of GGBFS will boost the density of both these mixtures, which may augment the thermal conductivity of the hardened AAP. Uysal et al. [121] and Asadia et al. [122] have noted that the disparities in context to the density of its constituent ingredients influence the thermal conductivity of concretes.

### 6.2. Residual Mechanical Strength after Elevated Temperature Exposure

The exploration by Huseien et al. [88] has extended the findings for the residual compressive strength and percentage of loss in weight of geopolymeric materials, whereby GBFS was substituted with FA at 0, 10, 20, 30, and 40 percentages, which exhibited the residual strength values augmented in line with the increasing percentages at rates of 18.2, 19.2, 28.1, 29.6, and 32.6%, correspondingly. Contrarily, the percentage loss in weight declined from 14.1 to 13.8%, 13.7%, 13.3%, and 13.5% when the quantity of FA was augmented from 0 to 10%, 20%, 30%, and 40%. Kovářík et al. [123] have put in plain words the thermomechanical attributes of particle-reinforced geopolymer composites with a variety of aggregate gradations of fine ceramic space filler. The outcomes have made it known that a greater quantity of fine ceramic particles under 90 mm and gradual distribution of other fractions from 150 to 710 mm leads to enhanced dimensional stability subsequently exposed to heat. Potassium-based geopolymers reinforced with fine ceramic particles divulged an unvarying flexural strength of 12 MPa and compressive strength of 90 MPa, both in an early state and following exposure at 1000 °C. According to Huseien et al. [21], the residual compressive strength encountered was directly proportional to the WCP quantity. Upon increasing WCP substituted GBFS from 50 to 70%, the values for residual strength were augmented from 17.7 to 40.1%, correspondingly. It is well known that the elevated temperatures lead to the speeding up of the procedure of the breakage of bonds in the geopolymer matrix [124]. The degree of damage and loss in strength appeared to be greater with specimens incorporating a higher level of Ca, the product of decomposition of CaCO_3_ (calcium carbonate) resulting in volume enhancement and causing the formulation of cracks [103]. During an experiment by Wang et al. [125], a Metakaolinite-based geopolymer synthesized through the process of geopolymerization displayed a good-quality mechanical strength [126]. 

### 6.3. X-Ray Diffraction (XRD) 

**Figure 3:**
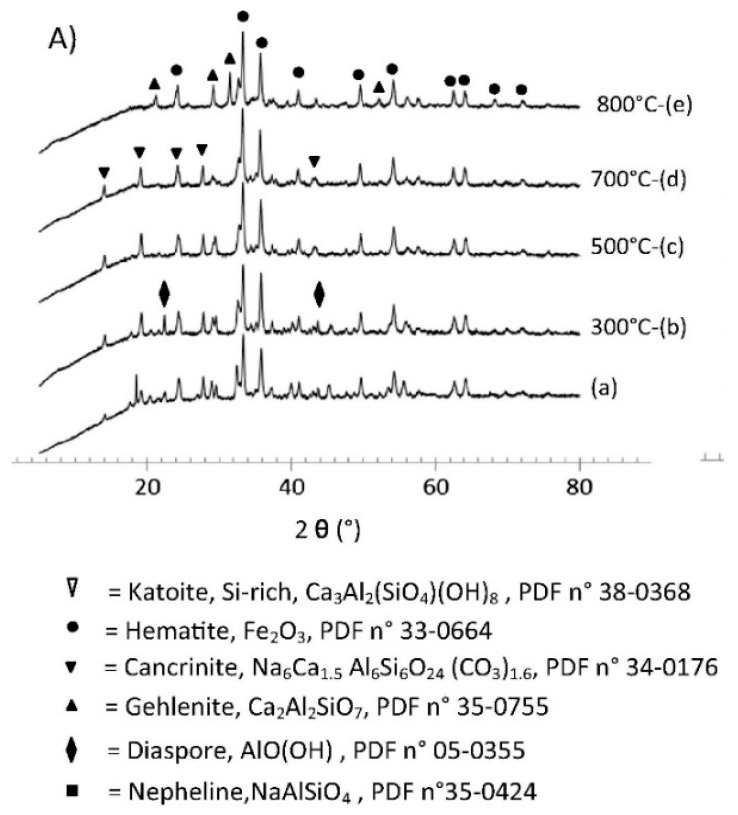
XRD analysis of (A) red mud.

Lemougna et al. [127] have described the synthesis and portrayal of the lower temperature of <800 °C ceramics manufactured from red mud geopolymer precursor. It was monitored based on the outcomes that the main crystalline phases existing in red mud are cancrinite and hematite and a small quantity of Si-rich, katoite, and diaspore. The hematite phase is not agitated to 800 °C, while the authors monitored a vanishing of the crystalline-type peaks linked to katoite and diaspore beyond 300 °C, and over 700 °C, cancrinite was observed. A new phase appears at 800 °C, gehlenite, probably formulated from the decomposition of cancrinite (Figure 3). Additionally, katoite was accounted to be present as a product of hydration in calcium-aluminate-cementitious materials [128,129].

**Figure 4:**
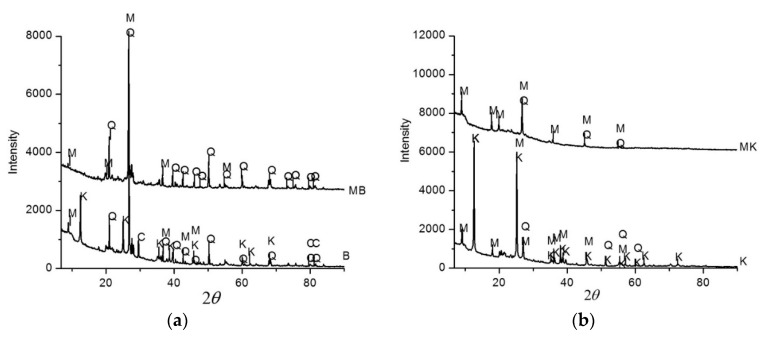
XRD patterns for (**a**) Sludge and (**b**) kaolin; M = mullite, K = kaolinite, Q = quartz, and C= calcite.

A few investigations on the thermal behaviour of red mud assigned the decomposition of cancrinite at a temperature lower than 900 °C to the CO_2_ emission, hematite composing the fundamental phase up to 1100 °C [130]. The existence of the initial crystalline phases in inorganic polymers manufactured from red mud, chiefly hematite and cancrinite, have also been accounted formerly [131,132,133], which is suggestive of the absence or lower dissolution of the abovementioned minerals in the alkaline state of the inorganic polymer synthesis. Following Belmokhtar et al. [134], XRD analysis disclosed that the mineral phases of the ceramic sludge and kaolin are equivalent. The crystalline nature of kaolinite in kaolin is shown to be higher than the ceramic industrial sludge used for testing. Consequently, the ceramic sludge waste(C) is therefore easier to dehydroxylate than the kaolin (K). The XRD investigation revealed that the ceramic sludge contains kaolinite. Nevertheless, the mineral content is lower than that of kaolin clay in ceramic sludge waste. In ceramic sludge waste, quartz (Q) and muscovite (M) are present after calcination (Figure 4), and their crystallines are somewhat decreased. The corresponding behaviour of the kaolin XRD is observed (Figure 4). The difference in the strength of reflection between the kaolin XRD pattern and the ceramic industrial sludge XRD pattern is due to the difference in the crystalline phase in every particle.

Kovářík et al. [123] have expressed the relative XRD patterns: MK largely made up of illite belonging to the monoclinic crystal system and quartz to the hexagonal system and a noticeable quantity of anorthic kaolinite and orthorhombic mullite. Additionally, tetragonal anatase and a trace quantity of rutile are present. The phase composition altered somewhat after the geopolymer was treated thermally at 1000 °C. In accordance with the XRD data, the kaolinite phase disintegrated entirely, and the quartz content was considerably decreased. No noteworthy disparities in the content of illite, mullite, titanium dioxides, anatase, and rutile were monitored. The said outcomes signify that during the thermal treatment at 1000 °C, no noticeable quantity of kalsilite or leucite crystalline phase formulated, contrary to previous findings for potassium-based geopolymers [135,136]. In the XRD pattern of metakaolinite employed in Wang et al.’s [125] experiment, it has been demonstrated that the metakaolinite was made up of an amorphous phase with a semicrystalline structure. There was little quartz and mullite present in the metakaolinite. The appearance of the broadband among 181 to 251 indicates that the geopolymer developed in the experiment was more or less entirely made up of an amorphous structure. Additionally, this points out that the quartz and mullite present in the metakaolinite did not participate in the reaction kinetics of geopolymerization. No palpable dissimilarity among the XRD patterns of the geopolymer and the calcined materials formed at 300 °C, 600 °C, and 900 °C was found; however, their bending strength was not the same. Akin to metakaolinite, these materials were all made up of an amorphous phase with a semicrystalline type structure. Significantly, the SEM image was of the calcined product at 900 °C. Hence, the conclusion can be made that the temperature of 900 °C was a significant transition temperature for the calcining course of action of the geopolymer developed for the experiment. At a temperature of less than 900 °C, the course of calcining could not make a noticeable structural modification in the geopolymer, although calcining declined the bending strength of the geopolymer. At 900 °C, the geopolymer was found partially melted and locally coagulated. Substantial interspaces marked their presence at 900 °C and removed the locally melted geopolymer. A smaller quantity of pores was available in the locally melted geopolymer. Barbosa et al. found that the K-geopolymer demonstrated a small sign of melting at 1400 °C [114], while the calcining temperature was elevated at 1200 °C; a material having a significantly higher crystallinity was developed. The investigation on XRD signified that the chief phase of the material was the mullite, which is an imperative mineral with brilliant heat resistant potential. The key phase of the resultant ceramic is mullite possessing a unique chemistry with the chemical formula 3Al_2_O_3_ 2SiO_2_. Still, there are a few impurities such as NaO, etc., found present in the ceramic. The melted geopolymer continued congealing, and interspaces condensed remarkably. While the temperature for calcining was elevated to 1500 °C, their key phase was still represented by mullite. However, the whole of the pores having a pore size of approximately10 μm was formulated at this temperature, i.e., a sort of mullite ceramic having a macroporous structure was formed. On the basis of this experiment, it was made known that complex-shaped metakaolinite-based geopolymers can effortlessly be manufactured through the technique applied. Huseien et al. [21] have represented the XRD patterns of geopolymer materials previous to and after elevating the temperature from 27 °C and raising it to 900 °C. Before and after the exposure of the early specimen to the elevated temperature of below 400 °C, an appearance of semicrystallized alumina silicate gel and quartz (Q) was encountered. The broader peaks of each geopolymer mortar component were visible in the region. Zeolites occurred as secondary reaction yields. This means they were formulated as the crystalline phase after the completion of a fire resistance test. The XRD patterns of geopolymer mortars were brought to exposure at 700 °C and 900 °C, in that order. The strong peaks in the WCP-based geopolymer largely identified the existence of quartz, nepheline, and mullite at the temperature of 700 °C. The only steady crystalline phase of the system of Al_2_O_3_–SiO_2_ was found as Mullite since it retained its strength both at room and elevated temperatures, proving its admirable stability even at higher temperatures with lower thermal expansion and resistance to oxidation. After exposure at 700 °C, quartz peaks continued to be stable, and the mullite peaks gradually turned out to be stronger. A phase transition from goethite to hematite happened at roughly 400 °C, whereby the majority of constitutional water molecules was discharged. The departing flux of hydroxide (OH) groups and concurrent diffusive reshuffle of the grain structure might provide grounds for the local accretion of in-house stress and may even be competent for fracturing the grains of hematite. At this particular temperature, the size and shape of a grain of the freshly developed hematite phase were still largely alike as those of the original goethite. When the exposure of the XRD pattern of a sample at 900 °C was made, the disclosed hematite started to vanish, whereas crystalline nepheline (AlNaSiO_4_-sodium aluminium silicate) was still present in the specimen while quartz remained the most important phase jointly with the mullite phase. In the context of a mortar containing 70% of WCP and a higher FA content, the peaks were steady at towering temperatures in comparison with other specimens. The investigation of Huseien et al. [88] has revealed the XRD patterns of geopolymeric materials incorporated with diverse levels of FA substituting GBFS, prior to and after the escalation of temperature from 27 to 900 °C. The existence of aluminosilicates gel and quartz having a semicrystalline form were monitored in the original sample both previous to and following the exposure to high temperatures of less than 400 °C. Aside from the test for fire resistance, the crystalline phase developed zeolites as a secondary reaction yield. At a temperature of 700 °C, the firm peaks in the geopolymeric WCP base, were decorated with the presence of mullite, quartz, and nepheline. At this time, mullite was also the single stable crystalline phase of the system of aluminosilicate, Al_2_O_3_-SiO_2_, by upholding the strength it had at room temperature, even after elevating the temperature to higher figures with a display of an inferior thermal expansion and resistance to oxidation. Upon exposure at 700 °C, quartz was found steady, and the enhancement in the strength of mullite was observed. Around 400 °C, the bulk of the molecules of the constitutional water were freed, and the phase altered from goethite to hematite. The simultaneous setting free of the OH groups of hydroxides took place with the modification of the grain structure, which could benefit by a stocking up of inner pressure that might be accountable for the fracture development in hematite grain structure. At this high temperature, the size and grain shape of the recently produced hematite phase harmonize to a great extent as the original goethite. While the specimen of XRD pattern was subjected to 900 °C, the initiation of the disappearing of the hematite phase occurred. Quartz retained the primary phase besides mullite along with the presence of crystalline nepheline—a sodium aluminium silicate having the chemical formula AlNaSiO_4_. The peaks exhibited stability at elevated temperatures when comparing them with the specimens made with samples *sans* FA and those containing 40% FA.

### 6.4. Scanning Electron Microscopy 

**Figure 5:**
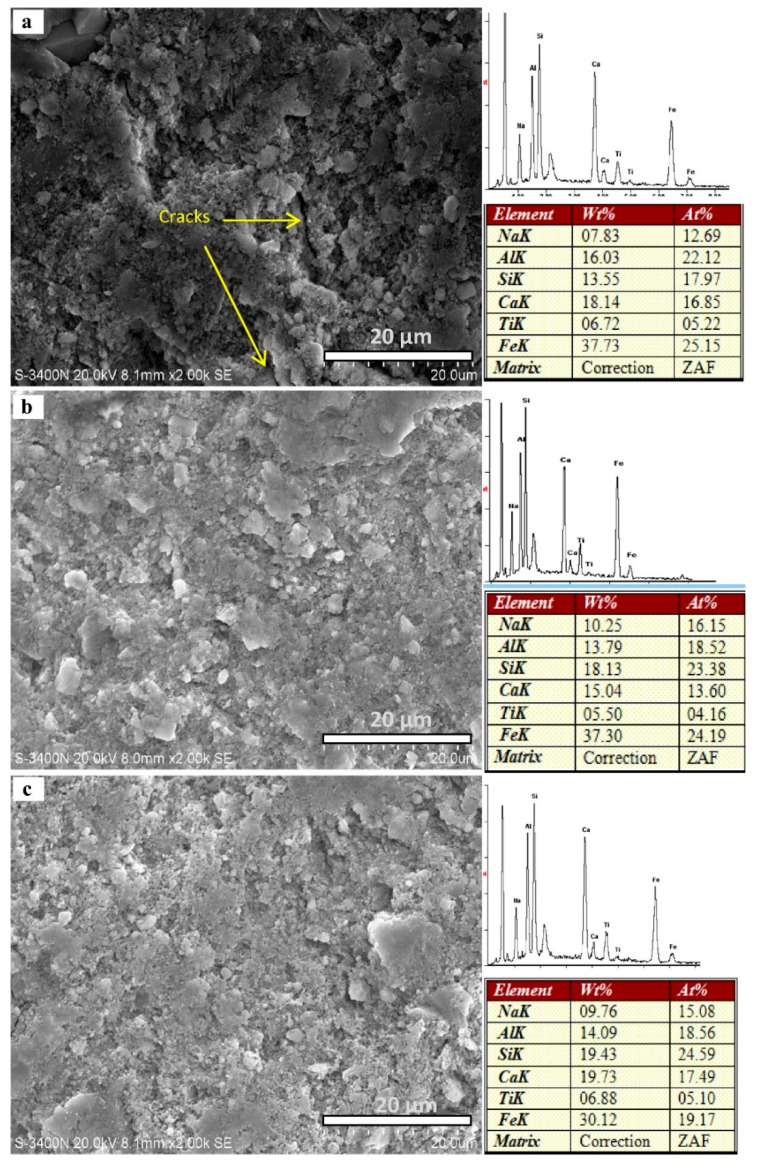
EDS analysis at (**a**) 60 °C (**b**) 300 °C and (**c**) 800 °C.

The SEM analysis by Belmokhtar et al. [134] on kaolinite containing geopolymeric materials has validated that the course of action of calcining does not adapt the morphology of the particles of kaolinite. Different particle sizes were observed in the obtained micrographs from the SEM study of ceramic sludge. In addition, the kaolinite particles as individual layers have broken borders and are covered by finer particles. On the surfaces of kaolinite particles, the smaller crystallites are simply found instead of attaching to the surface. The SEM investigation of ceramic industrial sludge micrographs showed more agglomerated particles than kaolin. The atmospheric absorption of H_2_O by ceramic waste is determined to be better as compared to kaolin. The same values were found in calcined ceramic sludge and a metakaolin SEM image. SEM from the calcinated ceramic sludge has shown that the quartz concentration is higher as compared to the metakaolin. Lemougna et al. [127] have carried out SEM analysis of red mud powder, which has revealed that many particles of red mud are very fine in size. The polished specimens have exhibited little similitudes in the microstructure of the inorganic polymer which has undergone curing for 28 days at 60 °C and its homologues postheated between 300 °C and 800 °C. The resemblances are possibly a result of the perseverance of lesser reactive phases, such as hematite, in this range of temperature in the geopolymer. The modification, which, with better transparency, was monitored in the microstructure evolution upon heating, is obvious through the alteration in the colour of the postheated specimen which turned out to be less dark in colour. Based on the weight and atom chemistry attained through EDX analysis, as depicted in Figure 5, it can be said that the chemical composition of the inorganic polymer mix is more or less homogenous at a microscopic scale. The heterogeneous type of distribution of elements was found on the EDX map, which is much more noticeable for iron and is instituted in harmony with the existence of nondissolved hematite in the XRD testing. More homogeneously distributed elements, viz., Si, Al, Na, and Ca, probably advance their participation in the inorganic polymer formulation. This finding conforms with a few previous investigations on the geopolymerization of diverse red muds [137,138] and hints that the inactivity of iron, more often than not in the form of hematite, is possibly a general fact during the geopolymerization of red mud.

Hairi et al. [138] have explored the role of iron through the geopolymerization of Canadian red mud and encountered a resemblance among the Mössbauer type spectra of the early materials of geopolymers, signifying that the Fe content, for the most part from hematite and goethite, in their cases, was predominantly present as an onlooker species rather than engaged in the structure of geopolymers. The SEM showed the metakaolinite-based geopolymer and its calcined yields at dissimilar temperatures. All the referred SEM images were taken at the cross-sections of specimens following the bending strength examination. It was found from Band C that no basic structure alteration was encountered when the geopolymer was calcined at the temperatures of 300 °C, 600 °C, and 900 °C. However, while the calcining temperature was augmented to 900 °C, local melting and integration was witnessed, although substantial interspaces were still found to be present. Since the temperature for calcining was accelerated incessantly, the melting level also perceptibly amplified accordingly. Additionally, the size and shape of the interspace have been modified with the boost in the temperature of calcining. At 1500 °C, macroporous material having an average pore size of roughly 10 μm was formulated. The material porosity reported was around 17%. The series of modifications in the morphology of the geopolymer has thrown light on the alternations of bending strength, apparent density, ratio of volume shrinkage, hardness, and escalation in the context of the temperature of calcining. Looking to the impact of the said morphology change, Novoselova et al. [139] have found that one sort of crack would have marked its presence in the products, while the geopolymer was sintered at an elevated rate of heating. However, here, there was no crack appearance at a heating rate of 150 °C/min. It was presumed that the root cause of this discrepancy is the chemistry of the activator and the technique for producing the geopolymer. The activator employed in the experiment was sodium hydroxide—NaOH—whereas Jia et al. have experimented with potassium hydroxide-KOH solution for activation. Furthermore, the wet mix was pressed under 4 MPa for 3 min to eliminate possible air bubbles entrapped in the yield on account of a stirring course. Huseien et al. have endeavoured to depict the outcomes of SEM micrographs of geopolymer materials containing a higher volume of WCP. The impact of the temperatures at 400 °C, 700 °C, and 900 °C on the structure of geopolymer materials incorporating 50%, 60%, and 70% of WCP was estimated. Denser structures of geopolymer materials were converted to lesser compact structures with a perceptible network of microcracks and bigger pores, which are more prominent with an enhancement in temperatures. The SEM micrographs display samples having 70% of WCP after exposing them to ambient temperatures of 400 °C, 700 °C, and 900 °C. The specimens have been imaged from a crashed section of mortars. Only a few microlevelled cracks were visible on the sample surface when exposed to a higher temperature. The particles of WCP with no participation in the reaction kinetics, FA, and a few sphere-shaped holes were observable. It is a well-known fact that FA possesses a considerable proportion of particles with hollow spheres and that is why when the hollow and spherical shaped particles are partly dissolved, they form strongly distributed smaller sized pores in the matrix. The said nonreacted particles were found to be present in hollow spaces, which might be due to the gap left behind by the dissolved particles of FA. On the contrary, the samples of 70% WCP have displayed much steadier surfaces at high temperatures in comparison with 50% WCP samples.

### 6.5. Fourier Transform Infrared Spectroscopy (FTIR)

Belmokhtar et al. [134] confirmed through FTIR research that a complete disposal of hydroxyl groups is caused by dehydrate in the ceramic sludge at an optimum temperature. The FTIR research showed that the cause for a structural modification in the context of kaolinites present in ceramic sludge, as observed in commercial kaolin, is dehydroxylation.

**Figure 6:**
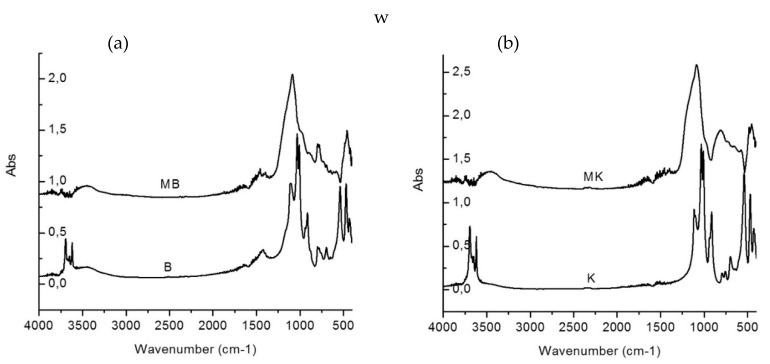
FTIR Spectra of (**a**) kaolin and (**b**) ceramic waste; MB = calcined ceramic waste, B = ceramic waste, MK = calcine kaolin, and K = kaolin.

In the FTIR spectrum (Figure 6), the kaolin and ceramic waste employed were immensely similar. There were two different absorption bands of the FTIR spectrum of noncalcined raw materials observed. Due to the extended hydroxyl vibrations, four bands are formed between 3700 cm^−1^ and 3600 cm^−1^. At the margins of kaolinite layers, the 3696 cm^−1^ band is ascribed to the hydroxyl-group vibration [140]. Due to the hydroxyl groups on the surface of the octahedral layers, the bands are 3668 cm^−1^ and 3653 cm^−1^. The layer is attributed to the inner hydroxyl groups at 3620 cm^−1^. The disappearance following the calcination of the referenced bands shows the elimination of most of the hydroxylic groups. The bands at 1104 cm^−1^ and 1019 cm^−1^ are transformed to an amorphous nature, characteristic to Si-O vibration in a small band positioned at about 1085 cm^−1^ following calcination. At 797 cm^−1^, the thin strip turned to a wide one. The bands are decreased in strength at 684 cm^−1^ and 539 cm^−1^. Additionally, there was a dislocation in the absorption band at 539 cm^−1^ at a higher frequency [140]. The abovementioned benefits indicate that the phenomenon of dehydroxylation modifies the kaolinite structure of the ceramic sludge, as in kaolin. Lemougna et al. [127] have found the large band at 1000 cm^−1^, which is ascribed to the stretching vibrations of Si (Al)–O groups, and it is quick to respond to the content of structural Si and Al [139]. It is monitored that the referred band becomes bigger after treatment of the specimen with a solution of Na_2_SiO_3_, probably suggestive of an augment in the amorphous content in the material. One more scrutiny is the comparatively immense dissimilarity in the shape of the band in the sample treated at 800 cm^−1^ in comparison with those treated at 60–700 cm^−1^, suggestive of a comparatively vital alteration in the structure of the material among 700 and 800 cm^−1^. The said modification is ascribed to the onset of solid-state reactions for the formulation of higher temperature mineral-like nepheline, as examined in the XRD section. The band around 1110 cm^−1^ crops up from the existence of the Si-O-Si bond [137]. Additionally, this band diminishes in the samples of inorganic polymers, suggestive of the replacement of Si–O–Si bonds with Si–O–Al bonds all through the formulation of the inorganic polymer setup [141]. The stretching vibrations of the Fe–O bands of the hematite structure are monitored around 450 and 550 cm^−1^. The band in the region of 1400–1500 cm^−1^ points towards the existence of O–C–O. It is recorded that the said band is gradually declined since the temperature accelerates [142]. The higher concentration of this band in the inorganic polymer treated at 60 °C is attributed to the existence of little structural water in the specimen. It is monitored that this band declines on heating but does not vanish entirely, the residual concentration at elevated temperature possibly cropping up from the water adsorption in the neighbouring air by the specimen, as frequently monitored in the cases of red mud or hematite samples [143]. The FTIR analysis by Wang et al. [125] exhibits IR spectrums of the geopolymer and the calcined yields under dissimilar temperatures. The IR spectrum of the metakaolinite employed in their experiment is also depicted. The broad adsorption bands on the IR spectra of the referred materials in the region of 1072 cm^−1^, 823 cm^−1^, and 457 cm^−1^ were ascribed to the Si–O stretching vibration, the Si–O–Al vibration, and the flexural vibration, correspondingly. The IR spectrum of the geopolymer differed from that of metakaolinite. Two broad bands in the region of 3420 cm^−1^ and 1680 cm^−1^ on the IR spectrum of the geopolymer pointed out that the geopolymer enclosed adsorbed atmospheric water. It could be found with no trouble, based on a comparison of these IR spectra, that the adsorbed atmospheric water could be eliminated by calcining. The FTIR spectrum by Huseien et al. [21] exhibits chief bands of 600 to 1200 cm^−1^ of specimens of geopolymers exposed to 900 °C. The FTIR shows band positions and matching band tasks for geopolymer materials containing a higher volume of WCP at 27 °C. The alterations in the band vibrations prior to and after exposing them to high temperatures were evidenced. The band frequency is enhanced with escalating temperatures and the decreased content of WCP and FA as well as diminishing GBFS quantity. In the cases of samples possessing 50% of WCP, the frequency of bands was augmented from 943.1 to 967.7 cm^−1^ with an escalating temperature from 27 to 900 °C, correspondingly. This monitored augment in the frequency of IR vibration was ascribed to the diminution of C (N)ASH gel in the geopolymer material setup, deteriorating the 3D structure in so doing.

### 6.6. Thermogravimetric and Differential Thermal Analyser (TGA)

Belmokhtar et al. [134] compared the TGA ceramic sludge pattern with kaolin and found that the kaolinite had a different dehydroxylating phase. In combination with two dissimilar gasses, the TGA pattern in ceramic sludge reflects two mass losses. The initial mass loss of around 4.07% begins with the emission of H_2_O vapours at 400 °C. The mass loss may be validated by kaolinite dihydroxylation, which marks its presence in the sludge, and the discussion of an amorphous natural phase called metakaolin. The second loss of mass begins at about 2.86% at 600 °C and is associated with CO_2_ emission, which is a result of calcite thermal breakdown in sludge [144,145].

**Figure 7:**
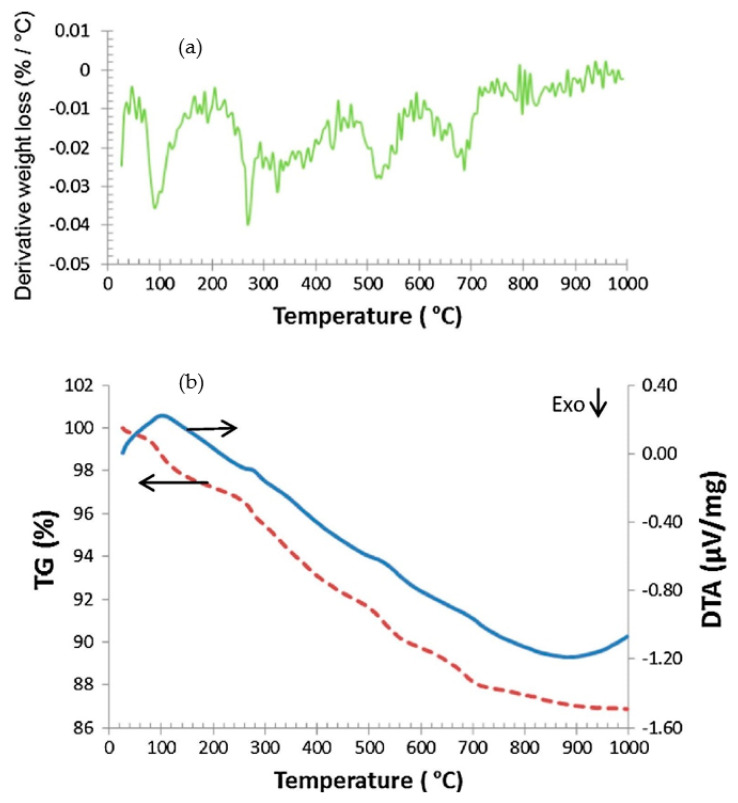
(**a**) DTA and (**b**) TGA analysis.

Lemougna et al. [127] have explained the topic in Figure 7, which exhibits the TG/DTA analysis and the derivative weight loss of the inorganic polymer. From this figure, it can be seen that the total weight loss following the heating of the inorganic polymer at 1000 °C is about 13%, almost half of the value achieved with the metakaolin-based inorganic polymer [146]. The derivative loss in weight signifies that the loss in weight on heating varies. There are four distinct regions of loss in weight. Based on the chemistry and the mineralogy of the specimen, it can be said that the loss in weight in the vicinity of 100 °C matches the loss of residual free water in the specimen. That in the region of 250–450 °C possibly crops up from the loss of structural water in the inorganic polymer and the decomposition of katoite and diaspore [129]. In contrast, that with around 500–600 °C is expected to happen from a partial discharge of CO_2_ from cancrinite and/or the decomposition of remaining structural water in the inorganic polymer, whereas that with something similar to 650–700 °C is attributed to the decomposition of cancrinite, which wholly vanishes beyond 700 °C, inconsistent with the X-ray analysis and formerly reported exploration on red mud. The specimens analysed by Kovářík et al. [123] have exhibited minimum dimensional alterations up to 150 °C, with total positive spreading out values in the range of 0.002 to 0.041%. The region of temperature is characterized by a smaller extent of expansion due to resistive dehydration of adsorbed water [147]. A remarkable shrinkage crops up in the second region of temperature over 150 °C, which is formed due to evaporation of water from the pores in the gel, leading to a collapse of micro- and nanopores [148]. The strain is caused by capillary shrinkage influenced by the discharge of adsorbed water and partial porosity reshuffling [149]. Subsequently, all specimens displayed a good-quality thermal stability in the temperature range of 300 °C and 800 °C. A trivial shrinkage at the commencement can mainly be attributed to the physical reduction of the gel on account of the sluggish dehydroxylation of bound hydroxyls. Huseien et al. [101] have illustrated the findings of the TGA curves of geopolymer materials possessing a higher volume of WCP. The impact of 400 °C, 700 °C, and 900 °C on the structure of 50%, 60%, and 70% of WCP samples was estimated. Denser structures of geopolymer materials were converted to the lesser compact structure with the perceptible setup of microcracks and bigger pores, which are more prominent with escalating temperatures. The TGA outcomes provide an idea about the loss in weight in the context of samples of geopolymers, where they declined with rising WCP substituted GBFS quantities of geopolymer materials. The values of loss in weight were reduced from 12.5 to 5.3% with increasing the WCP substituted GBFS from 50 to 70%, in that order. An analogous drift was encountered in TGA, as the FA substituted GBFS quantity augmented the loss in weight dropped. The amplified FA quantity as a substitution of GBFS in 50% of WCP samples from 0 to 30% led to a fall in loss of weight from 12.5 to 8.6%.

## 7. Conclusions

This review article reveals the competence of the reusability of ceramic wastes to develop green geopolymer composites. The following conclusions can be drawn:

- The application of ceramic aggregate as a substitution of natural sand has an optimistic result by fabricating higher compressive strength, flexural strength, and splitting tensile strength;
- The workability and setting time properties of alkali-activated mortars heightened with the upsurge in WCP content;
- Resistance against the elevated temperature of alkali-activated mortars was improved through the rise in WCP content;
- The substitution of GGBS by FA in the ternary assortments led to the lessening of deterioration up to 900 °C;
- The mortars incorporated with tile ceramic waste could provide a probable reapplication of waste material;
- Alkali-activated mortar incorporated a higher amount of FA and promoted the upgrade of the presentation of sulphate as well as acid resistance;
- The XRD examination reveals that the ceramic sludge contains kaolinite;
- From the TGA analysis of ceramic sludge and kaolin, it could be concluded that kaolinite is an inimitable phase able to be dehydroxylated;
- FTIR research shows that a structural modification of the kaolinite found in ceramic sludge is caused by a dihydroxylation process;
- The combination of geopolymers with ceramic waste is promising for lowering the energy and cost of materials in the construction sector.

## Data Availability

The data presented in this study are available on request from the corresponding author.
